# Effect of fluid and driving pressure on cyclical “on–off” flow of pulmonary microcirculation during mechanical ventilation

**DOI:** 10.1186/s40635-024-00689-6

**Published:** 2024-12-04

**Authors:** Siyi Yuan, Xiangyu Chen, Liangyu Mi, Yi Chi, Haoping Huang, Bo Liu, Chaofu Yue, Zeming Zhao, Longxiang Su, Yun Long, Şakir Akin, Can Ince, Huaiwu He

**Affiliations:** 1 Department of Critical Care Medicine, State Key Laboratory of Complex Severe and Rare Diseases, Dongcheng District, Peking Union Medical College Hospital, Peking Union Medical College, Chinese Academy of Medical Science, 1 Shuaifuyuan, Beijing, China; 2 Department of Critical Care Medicine, Affiliated Hospital of Jining Medical University, Jining, China; 3 Deparment of Intensive Care Unit, Qu Jing NO.1 Hospital, Qu Jing, Yun Nan China; 4 Jiamusi Central Hospital, Jiamusi, Heilongjiang Province China; 5 Department of Intensive Care, Erasmus MC University Hospital, Rotterdam, Netherlands; 6 Department of Intensive Care, Haga Teaching Hospital, The Hague, The Netherlands

## Abstract

**Objectives:**

This study aimed to identify the cyclical “on–off” flow of pulmonary microcirculation during inspiration and expiration by sidestream dark field imaging (SDF) technology in vivo and investigate the effects of volume status and driving pressure on cyclical “on–off” flow of microcirculation.

**Methods:**

24 ARDS-modeled rabbits were randomly divided into high-driving pressure group (HDP group) and low-driving pressure group (LDP group). Lung microcirculation measurements were performed using the SDF microscope at two timepoints (T1 CVP 2–4 mmHg, T2 CVP 8–10 mmHg). From T1 to T2, 10 ml/kg saline was infused to increase CVP. The cyclical “on–off” pulmonary microcirculation was quantitatively assessed by the change of microcirculation between expiration and inspiration.

**Results:**

Proportion of perfused vessels (PPV), microvascular flow index (MFI), perfused vessel density (PVD), and total vessel density (TVD) at expiration were significantly higher than inspiration in the HDP group. The HDP group has a higher ΔPPV and ΔPVD. After fluid loading, ΔPPV and ΔMFI decreased. TNF-α, IL-6, Ang-2, and vWF levels in the HDP group were higher. The HDP group also has a higher lung wet-weight/body weight ratio, lung wet-to-dry weight ratio, and more severe damage of pulmonary capillaries than the LDP group.

**Conclusions:**

The difference in alveolar perfused microcirculation between inspiration and expiration defined as cyclical “on–off flow” can be detected. High driving pressure can enhance the cyclical “on–off” flow, and fluid loading can relieve it. High driving pressure can potentially cause injury to pulmonary capillaries due to the phenomenon of “on–off” flow, thereby exacerbating ARDS.

**Supplementary Information:**

The online version contains supplementary material available at 10.1186/s40635-024-00689-6.

## Introduction

The impairment of pulmonary microcirculation is thought to play a pivotal role in the pathogenesis of acute respiratory distress syndrome (ARDS) [1, 2]. Katira et al. introduced the concept of cyclic “on–off” vascular flow, wherein pulmonary microcirculation perfusion diminishes during inspiration and intensifies during expiration [3]. Microvascular injury can be caused by flow interruption rather than elevated flow volume. However, existing research has been limited by method, with earlier studies relying on lung wet weight and ultrasound to assess pulmonary microcirculation [4, 5]. Consequently, the direct demonstration of the cyclic “on–off” flow phenomenon at the alveolar–capillary level remains elusive.

Prior investigations have indicated that both high driving pressure and hypovolemia can induce lung injury. Fougères et al. observed the collapse of pulmonary microvessels under low volume status, with reopening occurring upon increasing blood volume [6]. Other studies have associated high driving pressure with lung injury [7, 8] and the potential development of acute cor pulmonale (ACP) [9]. Nevertheless, the precise relationship between driving pressure and pulmonary microcirculation remains unclear, and the role of the cyclic “on–off” flow phenomenon in this mechanism warrants further exploration. Previous studies have lacked direct monitoring and dynamic assessment of pulmonary microcirculation perfusion at the alveolar–capillary level under varying volume statuses and driving pressures.

Sidestream dark field imaging (SDF) handheld vital microscopy [10] emerges as a valuable tool for evaluating pulmonary microcirculation perfusion in vivo [11]. Monitoring indicators such as the proportion of perfused vessels (PPV), microvascular flow index (MFI), and perfused vessel density (PVD) using SDF imaging enables direct observation and quantitative assessment of lung microcirculation, facilitating the identification of the cyclic “on–off” flow in perialveolar capillaries. Past studies have affirmed the efficacy of SDF imaging in monitoring pulmonary microcirculation in animal models [11].

This study aims to use SDF imaging to monitor end-inspiratory and end-expiratory perfusion of pulmonary microcirculation in a rabbit ARDS model. The objective is to visually validate the cyclic “on–off” flow phenomenon of alveolar capillaries and assess the impact of volume status and high driving pressure on the cyclic “on–off” flow in these capillaries.

## Methods

This study was approved by the Institutional Animal Care Committee of Peking Union Medical College Hospital. Twenty-four New Zealand White rabbits were anesthetized. Internal jugular venous catheters were inserted to measure central venous pressure (CVP). An arterial catheter was inserted into the internal carotid artery, and a Mostcare^®^ monitor (powered by PRAM; Vytech HealthTM, Padova, Italy) was used to measure cardiac output (CO) and PPV. Severe ARDS was induced by lung lavage followed by mechanical ventilation.

**Fig. 1:**
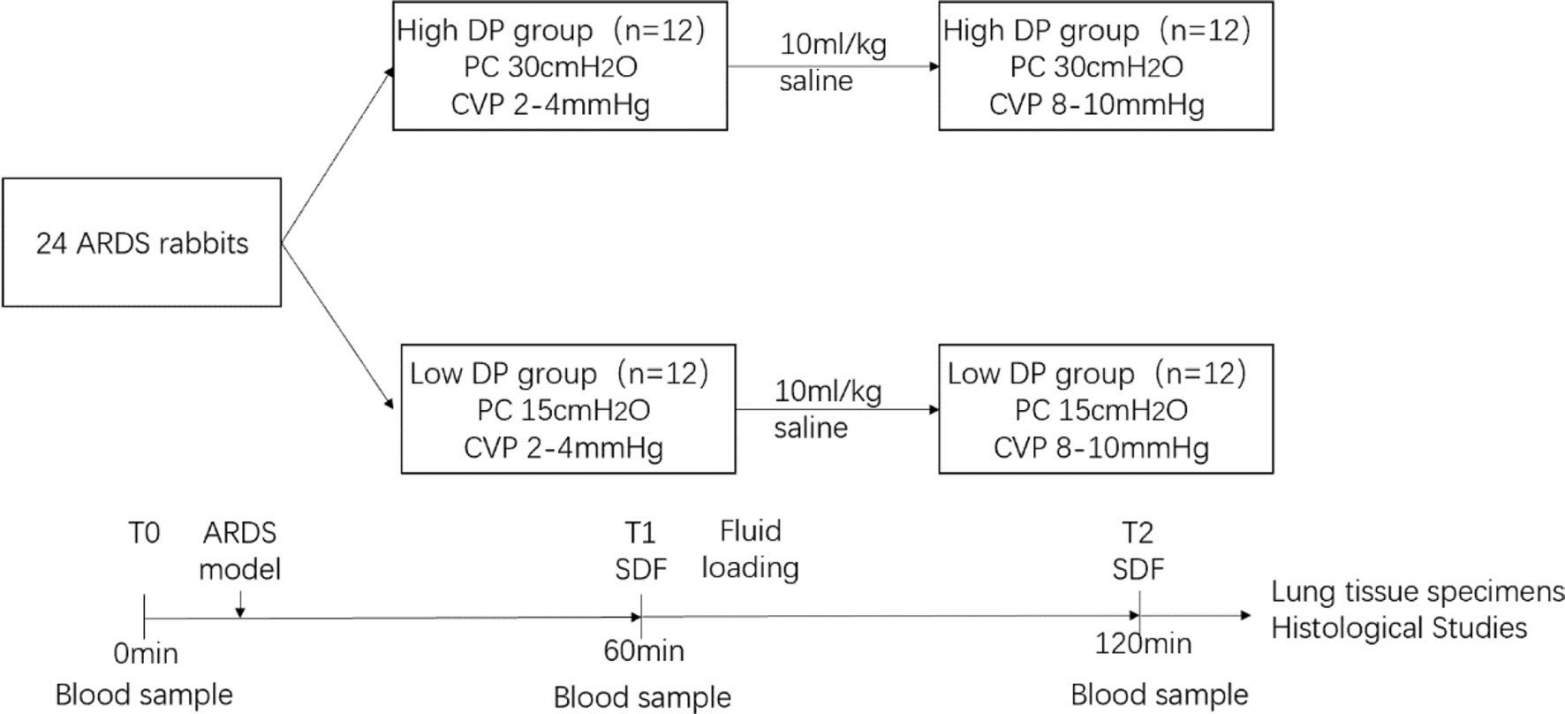
Experimental design. Twenty-four rabbits with acute respiratory distress syndrome (ARDS) were enrolled and stratified into two groups based on their received driving pressure (DP): a high DP group and a low DP group. Ventilation was performed using a pressure control model, with rabbits subjected to 30 cmH2O and 15 cmH2O driving pressures for 60 min. Following the initial ventilation period, a 10 mL/kg saline solution was administered via intravenous injection, leading to an elevation in central venous pressure from 2–4 mmHg to 8–10 mmHg. Subsequently, rabbits underwent an additional 60 min of ventilation. At the experiment's end, rabbits were sacrificed, and lung tissue specimens were collected for further analysis. Blood samples were obtained at three key timepoints: before the induction of ARDS, before fluid loading, and after 120 min of ventilation. ARDS, acute respiratory distress syndrome; DP, driving pressure; PC, pressure control; CVP, central venous pressure

Animals were then divided into two groups: (1) high driving pressure group (PC 30 cmH_2_O, PEEP 0 cmH_2_O) and (2) low driving pressure group (PC 15 cmH_2_O, PEEP 0 cmH_2_O) [5]. Animals were ventilated for 60 min at low volume status with pressure-controlled ventilation, then they underwent fluid loading. By administering approximately 10 ml/kg of fluid, the CVP was continuously monitored during the infusion process to achieve an elevation of CVP to 8–10 mmHg. The procedure is shown in Fig. 1.

### ARDS animal model

Rabbits were anesthetized and intubated to secure the airway. 10 ml of saline was injected directly into the bronchus via the tracheal tube, left to dwell for 10 s, and then aspirated. This injection process was repeated consecutively four times to achieve the desired degree of injury. Afterward, the rabbits were ventilated to restore oxygenation and prevent asphyxia. This procedure results in an acute increase in alveolar fluid, leading to impaired gas exchange, inflammation, and tissue damage, characteristic of ALI/ARDS. Consequently, after the process, the oxygenation index was consistently below 300 mmHg, meeting the criteria for ALI/ARDS.

### Measurement timepoints

Videos of subpleural pulmonary microcirculation were obtained from a fixed hole on the right chest wall by SDF microscopy, and hemodynamic data were collected from measurements. Pulmonary microcirculation and hemodynamic data were obtained at the following timepoints: (1) T1, baseline volume status, CVP 2–4 mmHg; (2) T2, after fluid loading, CVP 8–10 mmHg. Blood samples were collected at T0, before ARDS modeling; T1 and T2. The blood samples were used to measure the biomarkers, including tumor necrosis factor-α (TNF-α), interleukin-6 (IL-6), angiopoietin-2 (Ang-2), and von Willebrand Factor (vWF). After 2 h of ventilation, rabbits were killed, and lung samples were collected.

### SDF imaging and analysis

At each timepoint, the subpleural pulmonary microcirculation was evaluated at three different locations by MicroSee V100 system [Guangzhou Medsoft System Ltd., China (Medsoft System)]. The equipment is based on SDF imaging. The observation field is 0.73 mm × 0.55 mm, and the video frame rate is 30 fps with a resolution of 1.1 μm/pixel [12]. At each timepoint, three 10-s video clips were recorded at end-inspiration and end-expiration. The video clips used for analysis underwent a quality control test based on the quality of image resolution, image clarity, and elimination of pressure-induced artifacts. The Vascular Analysis software package (version 3.2; MicroVision Medical) was used for analysis according to expert consensus [13, 14]. The expected changes in MFI were divided into four categories, and each video was visually graded according to the following scale: 0, no flow; 1, intermittent flow; 2, slow flow (sluggish); and 3, continuous flow. The PPV is determined by calculating the percentage of perfused vessels relative to the total number of all vessels. Small vessels, specifically capillaries, are defined as being less than 20 µm in diameter. Total Vessel Density (TVD) is a software-based measurement of total vessel area per surface area. PVD is the percentage of perfused vessels × TVD [14].

### Evaluation of pulmonary edema

To quantify the degree of lung edema, the lung wet weight to body weight ratio and the lung wet/dry weight ratio were computed. In short, body weight and lung wet weight were measured after the harvested rabbit lung tissues were cut to exclude extrapulmonary structures. Subsequently, the right lung's middle lobe was removed and weighed (wet weight). After heating at 80 °C for 48 h, the lung sample was weighed again (dry weight). The ratio of lung wet to dry weight was computed.

### Statistical analysis

Unless otherwise noted, all data are expressed as mean SD or median (25th–75th percentiles). Paired data from different groups or timepoints were compared using paired-sample *t* test or Wilcoxon signed-rank test. All statistics were two-tailed, and a P value of 0.05 was considered significant. Statistical analyses were performed with SPSS 27 (IBM, Armonk, NY).

## Results

Twenty-four rabbits were anesthetized following the study protocol, with successful induction of ARDS models in all subjects. The rabbits were meticulously paired into 12 pairs based on age and weight and subsequently randomized into two groups: high driving pressure (HDP) and low driving pressure (LDP) groups. No discernible differences were observed between these groups concerning age, weight, and respiratory rate. However, a notable dissimilarity existed in tidal volume, with the LDP group exhibiting a significantly lower value compared to the HDP group (6.3 ± 0.68 ml/kg vs. 10.5 ± 0.85 ml/kg, *P* < 0.001).

### Hemodynamic parameters

As reported in Table 1, fluid loading significantly increased the CVP (P < 0.001) and decreased stroke volume variation (SVV) (*P* < 0.001) in both groups. Notably, in the HDP group, there was a significant rise in CO following fluid loading (*P* < 0.001), a response not observed in the LDP group. Moreover, mean arterial pressure (MAP) increased significantly in the HDP group after fluid infusion (*P* = 0.029).

**Table 1:** Circulatory parameters under different driving pressure and circulating volume status

		*Low-driving pressure group(N* = *12)*	*High-driving pressure group (N* = *12)*	*P value*
*CVP*				
	T1	3 ± 1.3	3.3 ± 1.3	0.608
	T2	7.8 ± 1.0^**#**^	8.1 ± 1.7^**#**^	0.612
*SVV*				
	T1	32.8 ± 4.4	45.0 ± 5.7^*^	< 0.001
	T2	24.8 ± 2.5^**#**^	32.8 ± 5.5^*#^	< 0.001
*CO*				
	T1	0.38 ± 0.06	0.36 ± 0.07	0.582
	T2	0.40 ± 0.06	0.44 ± 0.07^**#**^	0.240
*MAP*				
	T1	86.8 ± 3.5	82.8 ± 7.9	0.137
	T2	86.0 ± 6.5	88.7 ± 7.2^**#**^	0.461

Results showed that fluid loading significantly elevated CVP and decreased SVV in two groups, but CO and MAP changes were observed only in the high-driving pressure group. There was no change in CO and MAP with increased circulating volume in the low-driving pressure group. The high-pressure group exhibited higher SVV than the low-driving pressure group

^*^indicates a statistically significant difference between LDP group and HDP group

^#^indicates a statistically significant difference between T1 and T2

### Cyclic “on–off” flow of pulmonary capillaries

**Fig. 2:**
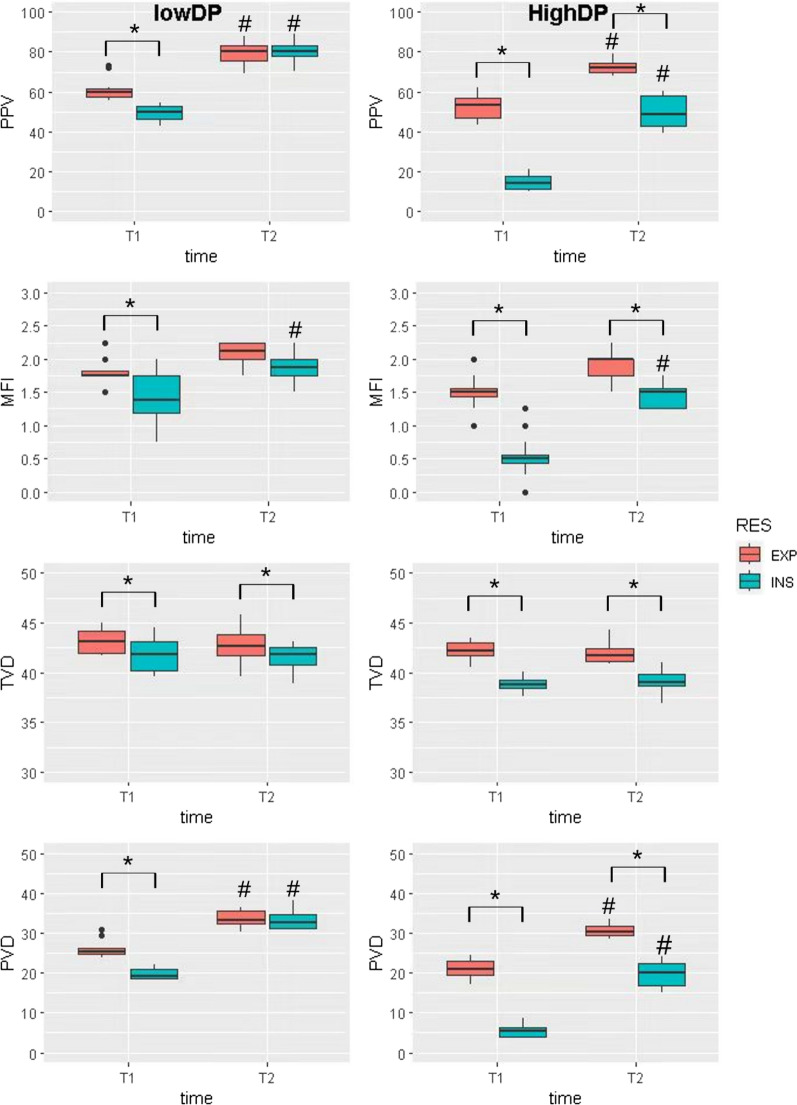
SDF parameters in two ventilation groups. SDF parameters, including PPV, MFI, TVD, and PVD, were assessed in two distinct groups. At the T1 timepoint, when both high and low driving pressure groups experienced low CVP, all indexes showed a significant increase during expiration compared to inspiration. After fluid loading at the T2 timepoint, ΔPPV, ΔMFI and ΔPVD (expiration–inspiration) narrowed both in the HDP and LDP group. PPV, proportion of perfused vessel; MFI, microvascular flow index; TVD, total vessel density; PVD, perfused vessel density. * indicates a statistically significant difference between inspiration and expiration. # indicates a statistically significant difference between T1 and T2

The comparison of SDF parameters, including PPV, MFI, TVD, and PVD, between inspiration and expiration in two groups is presented in Table 2 and illustrated in Fig. 2. The change between inspiration and expiration, denoted as ΔPPV, ΔMFI, ΔPVD, and ΔTVD, were detailed in Table 3. When evaluating SDF parameters at timepoint T1, it was observed that PPV (14.2 ± 3.2 vs. 53.6 ± 3.3, *p* < 0.001), MFI (0.50 ± 0.04 vs. 1.50 ± 0.06, *p* = 0.002), TVD (38.9 ± 1.1 vs. 42.2 ± 1.5, *p* < 0.001), and PVD (5.5 ± 1.3 vs. 21.1 ± 1.6, *p* < 0.001) significantly decreased more during inspiration than expiration in the high driving pressure group. PPV (50.0 ± 3.4 vs. 59.5 ± 1.9, *p* < 0.001), MFI (1.38 ± 0.37 vs. 1.75 ± 0.08, *p* = 0.007), TVD (41.8 ± 1.3 vs. 43.2 ± 0.9, *p* = 0.003), and PVD (19.3 ± 0.7 vs. 25.3 ± 0.6, *p* < 0.001) during inspiration was also smaller than expiration in the low driving pressure group at T1. Videos depicting the pulmonary microcirculation during both inspiration and expiration are provided in the Supplementary Materials. Videos 1–4 represent HDP at T1 during inspiration, HDP at T1 during expiration, LDP at T2 during inspiration, and LDP at T2 during expiration.

**Table 2:** SDF parameters under different driving pressure and circulating volume status

	T1			T2		
	EXP	INSP	p	EXP	INSP	*p*
PPV						
L	59.5 ± 1.9	50.0 ± 3.4^*^	< 0.001	80.2 ± 3.3	80.6 ± 4.1	0.85
H	53.6 ± 3.3^**#**^	14.2 ± 3.2^*#^	< 0.001	72.3 ± 2.2	48.7 ± 8.9^*#^	< 0.001
MFI						
L	1.75 ± 0.08	1.38 ± 0.37^*^	0.007	2.12 ± 0.12	1.88 ± 0.13	0.096
H	1.50 ± 0.06	0.50 ± 0.04^*#^	0.002	2.00 ± 0.25	1.50 ± 0.18^*#^	0.003
TVD						
L	43.2 ± 0.9	41.8 ± 1.3^*^	0.003	42.6 ± 0.11	41.8 ± 0.9^*^	0.002
H	42.2 ± 1.5	38.9 ± 1.1^*^	< 0.001	41.8 ± 0.07	39.0 ± 0.4^*^	0.004
PVD						
L	25.3 ± 0.6	19.3 ± 0.7^*^	< 0.001	33.1 ± 0.9	32.6 ± 1.3	0.469
H	21.1 ± 1.6	5.5 ± 1.3^*#^	< 0.001	30.5 ± 1.3	20.2 ± 2.7^*#^	< 0.001

At timepoint T1, it was observed that PPV, MFI, TVD, and PVD significantly decreased during inspiration than expiration in both groups. At T2, PPV, MFI, and PVD were still smaller during inspiration than expiration in the HDP group

PPV, proportion of perfused vessel; MFI, microvascular flow index; TVD, total vessel density; PVD, perfused vessel density

^*^indicates a statistically significant difference between expiration and inspiration

^#^indicates a statistically significant difference between LDP group and HDP group

**Table 3:** Change between inspiration and expiration under different driving pressure and circulating volume status

		T1	T2	*P*
ΔPPV				
	L	11.64 ± 5.42	− 1.06 ± 4.45^*^	< 0.001
	H	38.03 ± 3.92^#^	22.99 ± 5.53^*#^	< 0.001
ΔMFI				
	L	0.38 ± 0.27	0.21 ± 0.38	0.194
	H	0.96 ± 0.33^#^	0.40 ± 0.20^*^	< 0.001
ΔTVD				
	L	1.43 ± 1.19	1.31 ± 1.16	0.778
	H	3.29 ± 1.29^#^	2.97 ± 1.76	0.497
ΔPVD				
	L	6.12 ± 1.87	0.38 ± 2.11^*^	< 0.001
	H	15.54 ± 1.86^#^	11.07 ± 2.75^*#^	< 0.001

Fluid loading from T1 to T2 reduced the difference between inspiration and expiration of SDF parameters, but ΔTVD was unaffected. ΔPPV, ΔMFI, ΔTVD, and ΔPVD (expiration–inspiration) at T1 in the HDP group were substantially greater than in the LDP group

^*^ indicates a statistically significant difference between T1 and T2

^#^indicates a statistically significant difference between HDP and LDP groups

### Effect of driving pressure on cyclic "on–off" flow

At T1, during inspiration, PPV (14.2 ± 3.2 vs. 50.0 ± 3.4, p < 0.001), MFI (0.50 ± 0.04 vs. 1.38 ± 0.37, *p* < 0.001), and PVD (5.5 ± 1.3 vs. 19.3 ± 0.7, p < 0.001) were notably lower in the HDP group compared to the LDP group. At T2, although PPV (48.7 ± 8.9 vs. 80.6 ± 4.1, *p* < 0.001) and PVD (20.2 ± 2.7 vs. 32.6 ± 1.3, *p* < 0.001) during inspiration remained lower in the HDP group, the change between the two groups diminished. Additionally, ΔPPV (expiration–inspiration) at T1 in the HDP group was substantially greater than in the LDP group (38.03 ± 3.92 vs. 11.64 ± 5.42, *P* < 0.001). ΔMFI (expiration–inspiration) (0.96 ± 0.33 vs. 0.38 ± 0.27, *p* = 0.038), ΔTVD (expiration–inspiration) (3.29 ± 1.29 vs. 1.43 ± 1.19, *p* = 0.025) and ΔPVD (expiration–inspiration) 15.54 ± 1.86 vs. 6.12 ± 1.87 *p* < 0.001) had the same trend.

### Effect of fluid loading on cyclic “on–off” flow

Following fluid loading, there was a notable increase in PPV (14.2 ± 3.2 vs. 48.7 ± 8.9, *P* < 0.001), MFI (0.50 ± 0.04 vs. 1.50 ± 0.18, *P* = 0.003), and PVD (5.5 ± 1.3 vs. 20.2 ± 2.7, P < 0.001) during inspiration in the HDP group, accompanied by a decrease in ΔPPV (38.03 ± 3.92 vs. 22.99 ± 5.53, *P* < 0.001), ΔMFI (0.96 ± 0.33 vs. 0.40 ± 0.20, *P* < 0.001), and ΔPVD (expiration–inspiration) (15.44 ± 1.86 vs. 11.07 ± 2.75, P < 0.001). ΔPPV (11.64 ± 5.42 vs. -1.06 ± 4.45, *P* < 0.001) and ΔPVD (expiration–inspiration) (6.12 ± 1.87 vs. 0.38 ± 2.11, *P* < 0.001) also decreased after fluid loading in the LDP group. However, ΔTVD did not exhibit a significant difference after fluid loading in both groups.

### Markers of inflammation and vascular injury

**Fig. 3:**
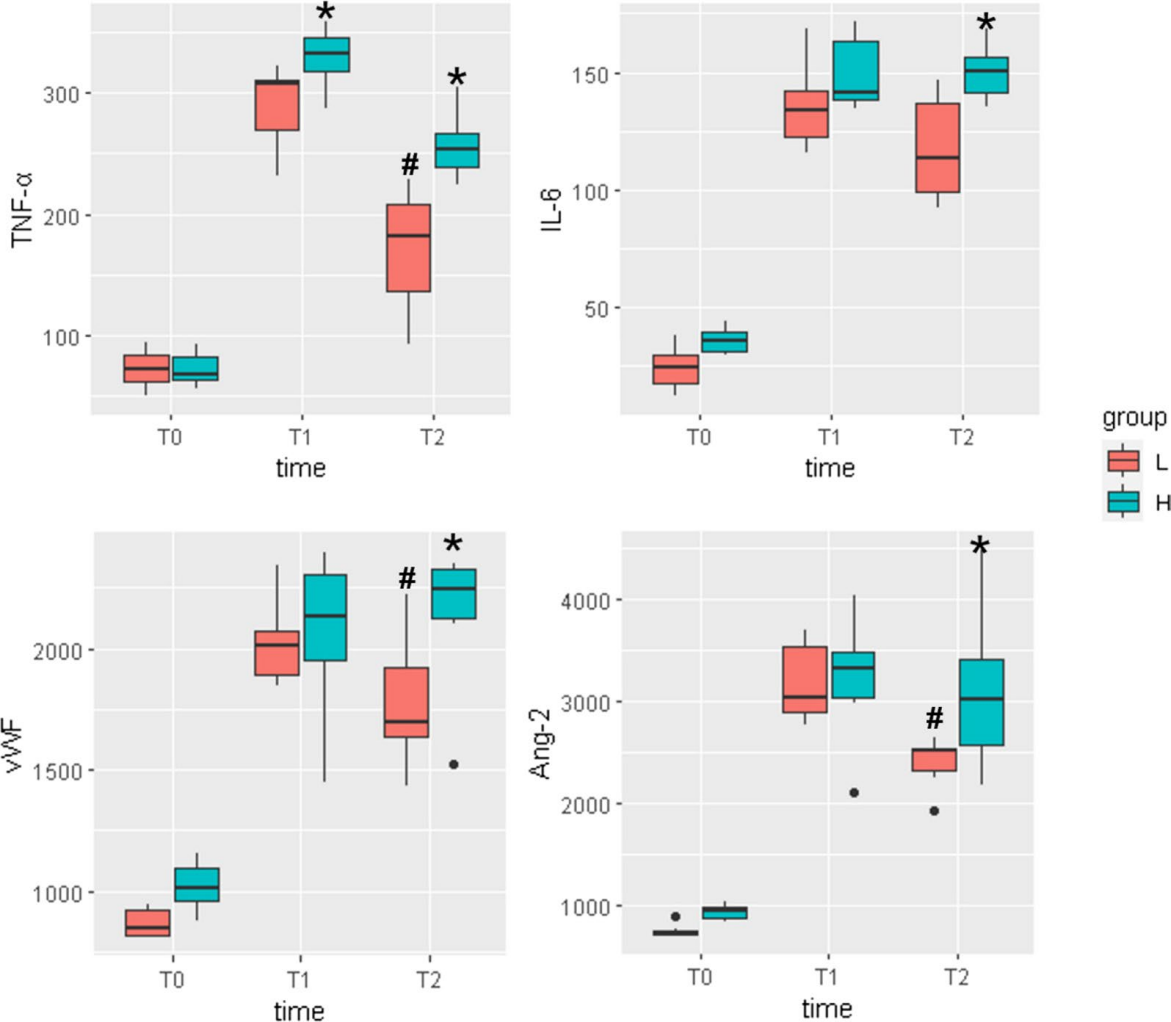
Cytokine profiling in response to ventilation strategies. The cytokine levels in two distinct groups were assessed at three critical timepoints during the experiment. Blood samples were collected at T0, before the induction of ARDS; T1, following 60 min of ventilation with low CVP; and T2, after an additional 60 min of ventilation with high CVP. The cytokines analyzed included TNF-α, IL-6, Ang-2, and vWF, with a focus on elucidating differences between the two groups. At the T1 timepoint, it was observed that the high DP group exhibited a significant elevation in TNF-α levels compared to the low DP group. However, no significant differences were noted in the other cytokines at this juncture. At the T2 timepoint, the high DP group displayed higher levels of TNF-α, IL-6,Ang-2 and vWF in comparison with the low DP group. In addition, in the LDP group, the levels of TNF-α, vWF, and Ang-2 were higher at T1 than at T2, while no significant difference was found between T1 and T2 in HDP group. TNF-α, tumor necrosis factor-α; IL-6, interleukin-6; Ang-2, angiopoietin-2; vWF, von Willebrand Factor. * indicates a statistically significant difference between LDP group and HDP group. **#** indicates a statistically significant difference between T1 and T2

We compared four biomarkers associated with inflammation and vascular endothelial injury, namely IL-6, TNF-α, Ang-2, and vWF, at three timepoints: T0 before ARDS induction, T1 at 60 min post-modeling, and T2 at 2 h post-modeling. The results indicated elevated biomarker levels at T1 and T2 compared to T0, affirming the efficacy of the modeling. Notably, at T2, the HDP group exhibited higher levels of IL-6 (151[141,157] vs. 113[99,137], *P* = 0.025), TNF-α (254[239,267] vs. 181[136,206], P = 0.006), Ang-2 (3016[2574,3409] vs. 2515[2316,2538], *P* = 0.049) and vWF (2242[2125,2330] vs. 1696[1632,1924], *P* = 0.037) than the LDP group, suggesting more severe inflammation and vascular endothelial injury in the former. In addition, in the LDP group, the levels of TNF-α, vWF, and Ang-2 were higher at T1 than at T2. The detailed biomarker results are depicted in Fig. 3.

### Pulmonary edema

**Fig. 4:**
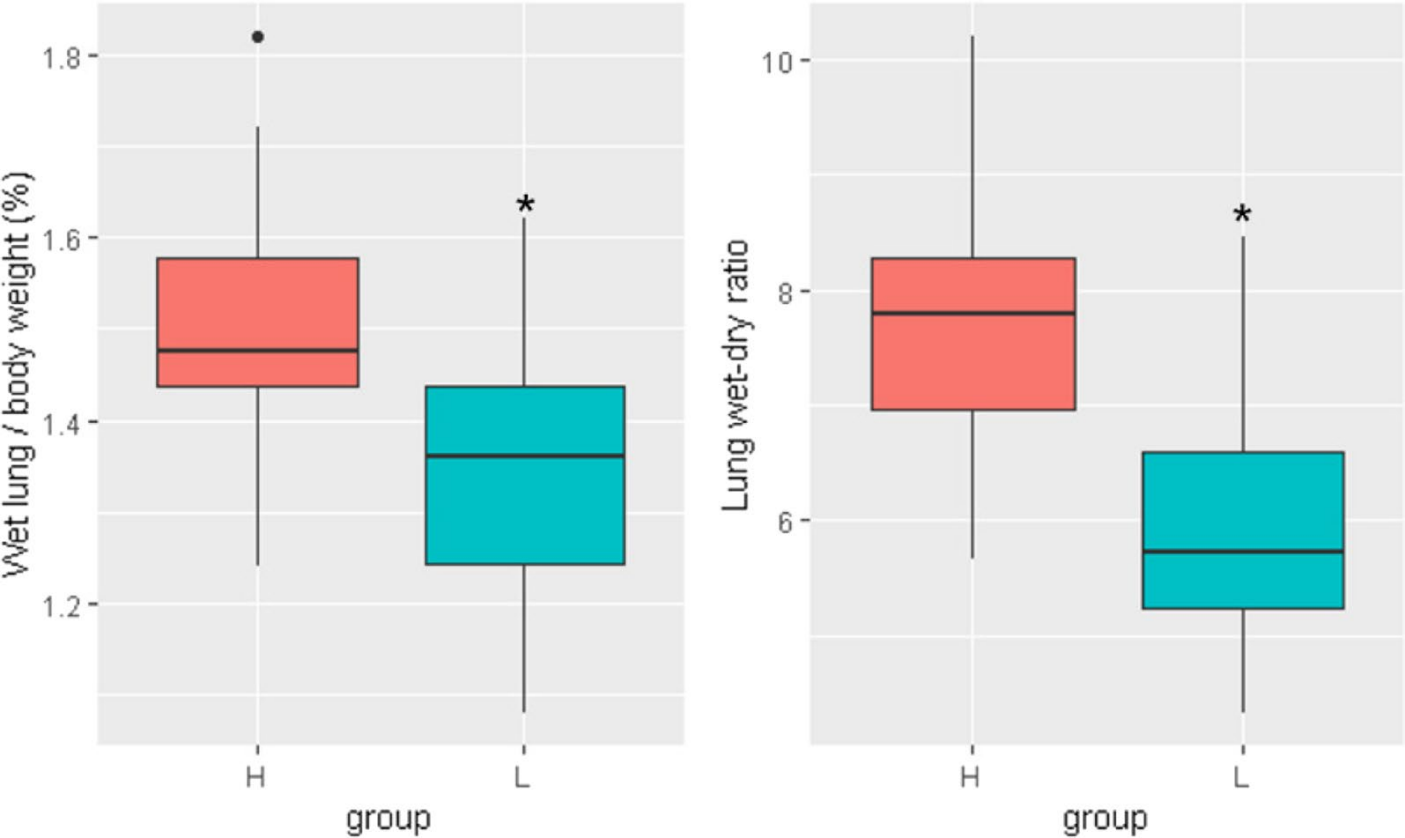
Pulmonary edema comparison. Rabbits subjected to low DP group exhibited a significantly lower proportion of wet lung/body weight and lung wet–dry ratio than those exposed to high DP. * indicates a statistically significant difference between the LDP group and the HDP group

Pulmonary edema, represented as the ratio of the lung wet weight to the body weight and the lung wet-to-dry weight ratio, revealed significant differences between the HDP and LDP groups. The comparison of pulmonary edema is shown in Fig. 4. The HDP group exhibited a notably higher lung wet weight/body weight ratio than the LDP group (1.48 ± 0.07 vs. 1.36 ± 0.09, P = 0.019). Similarly, the lung wet-to-dry weight ratio was higher in the HDP group (7.78 ± 0.49 vs. 5.72 ± 0.58, *P* = 0.007).

## Discussion

This study had three important findings as follows: (1) the cyclic “on–off” flow of pulmonary microcirculation could be demonstrated using SDF imaging at the level of alveolar capillaries, which means that pulmonary microcirculatory perfusion decreases during inspiration and increases during expiration. (2) The cyclic “on–off” flow was influenced by driving pressure. High driving pressure may exacerbate this phenomenon. (3) Fluid loading may decrease the cyclic on–off flow of the pulmonary microcirculation.

### Cyclic “on–off” flow of the pulmonary microcirculation

Cardiopulmonary interactions have been demonstrated in previous studies [15]. Mechanical ventilation may cause increased intrathoracic pressure, affecting venous return, resulting in decreased right ventricular preload and increased afterload, resulting in decreased right cardiac output. Tabuchi et al. reported the first direct visualization of alveolar pendular airflow [16]. In addition, Kavanagh et al. suggested that high tidal volumes and zero PEEP could cause cycles of obliteration of perfusion during inspiration and increased perfusion during expiration [3]. Similar results were found in two other studies [4, 17]. However, these studies only evaluated the right ventricle's cyclic “on–off” flow by ultrasound. Few studies have directly observed the behavior of the pulmonary microcirculation. In this study, we directly visualized alveolar capillary perfusion using SDF imaging and found that the pulmonary microcirculation varies with the respiratory cycle. In the high driving pressure group, PPV and MFI decreased during inspiration and increased during expiration, which means that the perfusion of the pulmonary microcirculation decreased during inspiration and recovered during expiration. In this study, we identified the cyclic “on–off” flow of the pulmonary microcirculation at the level of alveolar capillaries by SDF.

The mechanism behind this cyclic “on–off” flow phenomenon may be similar to that of the West's zone. High driving pressure increases intra-alveolar pressure, and low volume status can decrease hydrostatic pressure within alveolar capillaries to exacerbate the cyclical “on–off” flow in the alveolar capillaries by causing the intra-alveolar pressure in certain regions to exceed the intracapillary pressure from West Zone 3 to Zone 2 or even Zone 1 during inspiration.

### High driving pressure exacerbates cyclic “on–off” flow

It is widely accepted that high driving pressure can increase the morbidity of ARDS patients [18, 19]. The mechanism is always related to the damage of alveolar epithelial cells by high driving pressure. Furthermore, transmural pulmonary vascular pressure fluctuations and pulmonary microcirculation disturbance may also play a role in the adverse effect of high driving pressure. This study found that pulmonary microcirculation perfusion during inspiration was lower in the HDP group than in the LDP group. In addition, ΔPPV and ΔMFI between inspiration and expiration were greater in the HDP group. The cytokine and histopathologic results also showed that the HDP group had more severe lung injury than the LDP group. This result indicated that high driving pressure may affect the pulmonary microcirculation and exacerbate the cyclic “on–off” flow in alveolar capillaries. Cyclic flow interruption, rather than simply high flow, may cause flow-mediated microvascular injury and worsen the prognosis of ARDS patients.

### Fluid loading improves cyclic "on–off" flow

In this study, we found that alveolar capillary perfusion increased after fluid loading. In addition, the difference between end-inspiratory and end-expiratory perfusion also decreased after fluid loading, which means that fluid loading may diminish the cyclic “on–off” flow of the pulmonary microcirculation. Despite our experimental results, fluid loading in ARDS patients may have adverse effects, such as increased hydrostatic pressure and pulmonary edema. Currently, the management of ARDS aims to maintain the minimum volume status while maintaining macrocirculatory perfusion [20]. Notably, the cyclic “on–off” flow of pulmonary capillaries in the low-volume state may be a potential risk for lung injury. Previous research has shown that fluid loading can effectively counteract the collapse of alveolar microcirculation and improve alveolar microcirculation [6]. Our results support this finding. However, our study did not independently investigate the role of volume status, and further investigation is needed to determine the effect of volume status on cyclic “on–off” flow and lung injury.

### Cyclic “on–off” flow and lung injury

Only a few specific biomarkers have a high specificity and rapid response to detect vascular endothelial injury [21, 22]. Pulmonary vascular injury should be considered when setting respiratory parameters and fluid administration in patients with ARDS. In this study, inflammatory factors IL-6 and TNF-α and biomarkers of vascular endothelial injury Ang-2 and vWF were increased in the high driving pressure group. The HDP group exhibited a higher lung wet weight/body weight ratio and wet-to-dry weight ratio than the LDP group. The results indicate that high driving pressure may cause lung injury, especially capillary injury. Cyclic “on–off” flow may play a role in this mechanism. The study investigated the effects of two factors, volume and driving pressure, on microcirculation, which may interact in a confounding manner. The attenuation of the cyclic on–off flow phenomenon and the reduction of inflammatory factors following volume expansion in the LDP group are consistent. In contrast, this effect was not observed in the HDP group, possibly due to the increased vascular permeability caused by greater vascular injury in this group. At this point, fluid loading may act as a double-edged sword in the HDP group, improving the cyclic on–off flow phenomenon while simultaneously leading to increased capillary leakage. Fluid loading appears to be beneficial for the LDP group, but for the HDP group, it may have both beneficial and detrimental effects.

### Translational relevance of cyclic “on–off” flow

We have provided a method for evaluating pulmonary vascular injury by comparing the differences in pulmonary microcirculatory perfusion between end-inspiration and end-expiration, which serves as an important reference for assessing mechanical ventilation-related pulmonary vascular injury in future animal experiments. In addition, this study found that the cyclic on–off flow phenomenon is associated with pulmonary vascular injury under high driving pressure. Although it is not feasible to monitor the SDF in humans, efforts can be made to identify biomarkers related to the cyclic on–off flow phenomenon to monitor pulmonary vascular injury during high driving pressure mechanical ventilation. ARDS management often pursues a low volume status, which may exacerbate the cyclic on–off flow phenomenon, leading to pulmonary vascular injury. Previous studies have also suggested that a low volume status may cause the collapse of alveolar capillaries, affecting alveolar microcirculation[6]. The volume management in ARDS should consider the impact of cyclic on–off phenomena on pulmonary microcirculation.

### Limitations

This study has several limitations. First, we did not use ultrasound or SG catheters to monitor right ventricular function and pulmonary hypertension. Therefore, the effect of cyclic on–off flow on RV function and pulmonary vascular resistance was unclear. Second, the observation of cyclic on–off flow here is relatively generalized and only in the peripheral lung vessels. There is a difference in pulmonary microcirculatory perfusion between the peripheral and central regions of the lung in ARDS. Due to the complexity of alveolar microcirculation and the difficulty of manipulation, we have focused on the changes in microcirculation perfusion in a region of interest during the respiratory cycle to estimate this phenomenon. Future research is needed to investigate the periodic changes in a fixed microcirculation unit to understand this phenomenon better. Third, the duration of mechanical ventilation is only 2 h, which is relatively short. However, the inflammatory factors and microscopic results showed that 2 h was enough to cause ventilation-induced lung injury in this study. As our methodology only allowed for tissue sampling and measurement post-mortem in the rabbit model, the pulmonary edema is influenced by the effects of fluid resuscitation. Fourth, when comparing end-inspiratory and end-expiratory capillary density, it is inevitable that the alveolar size will be affected. Fifth, the current study solely explores the impact of driving pressure on the cyclic on–off flow phenomena, while PEEP may also play a role in this process. The effects of PEEP need to be further investigated. Sixth, using SDF imaging requires a chest opening operation, which will affect the pressure in the chest cavity. We used small window technology to reduce the diameter of the chest wound as much as possible to reduce the impact on intrathoracic pressure. Finally, the application of SDF imaging to pulmonary microcirculation monitoring has not yet been widely used, and the measurement of some indicators still lacks uniform standards.

## Conclusion

This study has demonstrated the cyclic “on–off” flow of pulmonary microcirculation in rabbit models of ARDS. High driving pressure may cause the cyclic “on–off” flow, and fluid loading may relieve it. High driving pressure can potentially cause injury to pulmonary capillaries due to the phenomenon of “on–off” flow, thereby exacerbating ARDS. Although fluid loading may reduce the intensity of this phenomenon, its significance in preventing pulmonary vascular injury in ARDS is yet to be studied.

## Supplementary Information

Additional file 1.

Additional file 2.

Additional file 3.

Additional file 4.

Additional file 5.

## Data Availability

Materials can be found in the Supplementary Materials.
