# Supplementary material for: Effect of fluid and driving pressure on cyclical “on–off” flow of pulmonary microcirculation during mechanical ventilation
Source: Intensive Care Med Exp. 2024 Dec 4;12:112. doi: 10.1186/s40635-024-00689-6 (PMC11618265; doi:10.1186/s40635-024-00689-6)
Supplement: Supplementary file 1 — Additional file 1. [file 40635_2024_689_MOESM1_ESM.docx]

**Supplementary Materials**

Video 1 presents the sidestream dark field (SDF) recorded images at the end of inspiration for the high driving pressure group (PC 30 cmH_2_O, PEEP 0 cmH_2_O) before fluid loading(CVP 2-4 mmHg). It can be observed from the video that some alveoli have ruptured and coalesced into larger alveoli, with a low density of effectively perfused capillaries in the field of view and slow, sluggish blood flow.

Video 2 presents the SDF recorded images at the end of expiration for the high driving pressure group (PC 30 cmH_2_O, PEEP 0 cmH_2_O) before fluid loading (CVP 2-4 mmHg). The video reveals that the alveoli are significantly smaller at the end of expiration compared to the end of inspiration, with an increased density of effectively perfused capillaries in the field of view. The blood flow velocity is faster than in Video 1, yet still relatively slow, and there is discontinuity present.

Video 3 presents the SDF recorded images at the end of inspiration for the low driving pressure group (PC 15 cmH_2_O, PEEP 0 cmH_2_O) after fluid loading (CVP 8-10 mmHg). The video shows that the alveoli are of normal size with no significant damage, and there is a higher density of effectively perfused capillaries in the field of view, with only a few instances of sluggish and discontinuous blood flow.

Video 4 presents the SDF recorded images at the end of expiration for the low driving pressure group (PC 15 cmH_2_O, PEEP 0 cmH_2_O) after fluid loading (CVP 8-10 mmHg). The alveoli are smaller than in Video 1, and the field of view contains a higher density of effectively perfused capillaries. The blood flow is rapid and continuous.

The accompanying schematic diagrams depict the alveoli and the blood flow in the capillaries surrounding the alveoli within the field of view.


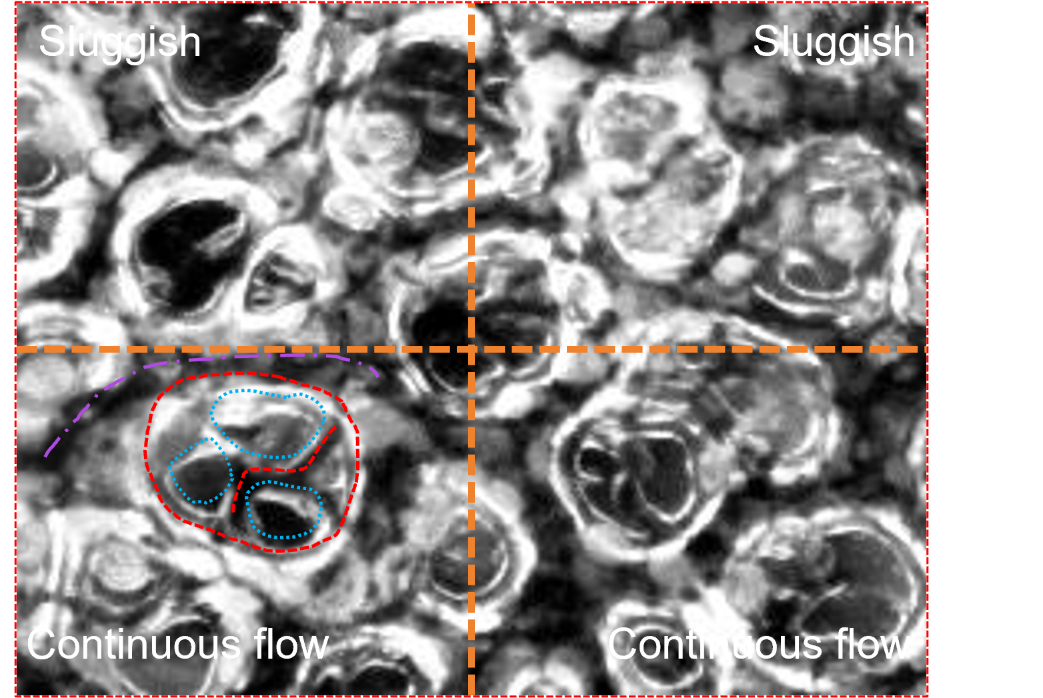


Figure S1 The schematic figure of the SDF microvascular indices.

The alveolar contours are outlined with blue dashed lines, and the blood flow in the alveolar capillaries is indicated by red dashed lines. Broken purple lines indicate extra-alveolar microvessels. The moving black dots visible in the videos represent red blood cells.

1. Microvascular Flow Index (MFI): The SDF video was divided into four quadrants according to the standard cross method as illustrated in the diagram. Each quadrant was visually graded using the following scale: 0 for no flow, 1 for intermittent flow, 2 for slow flow (sluggish), and 3 for continuous flow. The MFI is calculated as the average of the scores from the four quadrants. In this instance, the grading for the four categories was (2, 2, 3, 3), resulting in an MFI of 2.5.
2. Proportion of Perfused Vessels (PPV): The PPV is determined by calculating the percentage of perfused vessels relative to the total number of all vessels. Small vessels, specifically capillaries, are defined as being less than 20 µm in diameter. As shown in the illustration, a white highlight represents an alveolar unit, with blue dashed lines indicating three alveoli and red dashed lines representing the surrounding capillaries. The blood flow velocity in the vessels is graded and labeled whether they are perfused vessels. This method is applied to map all capillaries around the alveoli in the field of view, and the PPV is calculated as the percentage of perfused vessels relative to the total number of all vessels.
3. Total Vessel Density (TVD): All capillaries within the alveolar units are delineated, and the density of capillaries per unit area of the field of view is calculated. The TVD is measured using software-supported measurement of the total vessel area per surface area, which is a determinant of capillary distance (diffusive capacity).
4. Perfused Vessel Density (PVD): The PVD is calculated as a percentage of perfused vessels × TVD. The PVD serves as a determinant of capillary distance (diffusive capacity) and red blood cell velocity (convective capacity).
